# Metastatic INI-1−deficient undifferentiated lung cancer with EGFR 19del mutation identified in pleural effusion: a case report and review of the literature

**DOI:** 10.3389/fonc.2025.1539309

**Published:** 2025-04-11

**Authors:** Zhen Guo, Tianyuan Li, Xiufang Duan

**Affiliations:** ^1^ Department of Radiation Oncology, Liaocheng People’s Hospital, Liaocheng, Shandong, China; ^2^ Department of Respiratory, Shenxian People’s Hospital, Liaocheng, Shandong, China; ^3^ Department of Oncology, Shenxian People’s Hospital, Liaocheng, Shandong, China

**Keywords:** INI-1, lung cancer, EGFR 19del mutation, SWI/SNF complex, INI-1-deficient undifferentiated lung cancer, pleural effusion

## Abstract

INI-1 is a core component of the switch/sucrose-non-fermenting (SWI/SNF) complex, an ATP-dependent chromatin remodeling complex that plays a critical role in DNA repair, transcriptional regulation, and cellular differentiation. Intrathoracic tumors driven by INI-1 inactivation are exceptionally rare. This report presents the first documented case of metastatic INI-1-deficient undifferentiated lung cancer harboring a co-occurring epidermal growth factor receptor (EGFR) exon 19 deletion mutation. The clinical and pathological characteristics of the tumor are described, followed by a comprehensive review of the relevant literature.

## Introduction

INI-1 (also known as SNF5, SMARCB1, and BAF47) is a tumor suppressor gene located on chromosome 22q11.2, with expression detected in nearly all human tissues (1). It exerts its tumor-suppressive function through multiple pathways, including p16-mediated cell cycle regulation and inhibition of MYC target activation, thereby playing a crucial role in tumor suppression (2). Additionally, INI-1 is a core subunit of the switch/sucrose-non-fermenting (SWI/SNF) chromatin remodeling complex, a member of the ATP-dependent chromatin remodeling complex family. These complexes utilize ATP hydrolysis to alter chromatin architecture by modulating nucleosome positioning, eviction, insertion, and exchange (3–5). Consequently, they regulate the accessibility of genomic regions to transcriptional machinery, DNA-binding proteins, cofactors, and other regulatory elements, thereby orchestrating gene expression and DNA repair.

Chromatin remodeling complexes are categorized into four major families based on subunit composition and biochemical activity: SWI/SNF, INO80/SWR1, ISWI, and NURD/CHD. Among these, the SWI/SNF complex is most strongly implicated in tumorigenesis (4). These complexes are evolutionarily conserved, with homologous structures identified from yeast to mammals (4). In mammals, the SWI/SNF complex is further classified into three subfamilies: canonical BAF (cBAF), polybromo-associated BAF (PBAF), and the recently identified non-canonical BAF (ncBAF) (6). While all three subfamilies share core subunits, including SMARCC1, SMARCC2, SMARCD, SMARCA4, SMARCA2, and SMARCB1 (INI-1), distinct subunits confer unique functional properties. For example, ARID1A and ARID1B are exclusive to the cBAF complex, whereas ARID2, PBRM1, and BRD7 are specific to PBAF (7).

Genes encoding SWI/SNF complex components are mutated in nearly 25% of all human cancers, making them the most frequently altered chromatin-related cancer genes (6–8). In lung cancer, mutations in SWI/SNF subunit genes occur in approximately 20% of cases, with SMARCA4, ARID1A, ARID2, and SMARCA2 being the most commonly affected (9). The 5th edition of the WHO classification of thoracic tumors recognized SMARCA4-deficient undifferentiated tumors as a distinct entity, characterizing them as malignant neoplasms with an undifferentiated or rhabdoid phenotype resulting from SMARCA4 (BRG1) deficiency (10). Furthermore, SMARCA4 loss has been identified in approximately 3–6% of NSCLCs (11). In contrast, INI-1-deficient lung cancers are exceptionally rare, with only eight cases documented to date. Among these, two cases exhibited distinct molecular alterations: one with copy number variations in EGFR and another with STK11 loss. No other recurrent pathogenic mutations, including those in KRAS, RET, ROS1, EGFR, or ALK, were identified (12–18).

This research describes the cytological and immunohistochemical (IHC) characteristics of an SMARCB1 (INI1)-deficient intrathoracic neoplasm detected in pleural fluid. Next-generation sequencing (NGS) panel 16 analysis identified an EGFR exon 19 deletion mutation, marking a notable genomic alteration in this tumor. To date, no documented cases have reported the INI1 deficiency and a canonical EGFR exon 19 deletion mutation. This finding holds significant implications for both pathologists and clinicians.

## Case presentation

**Figure 1:**
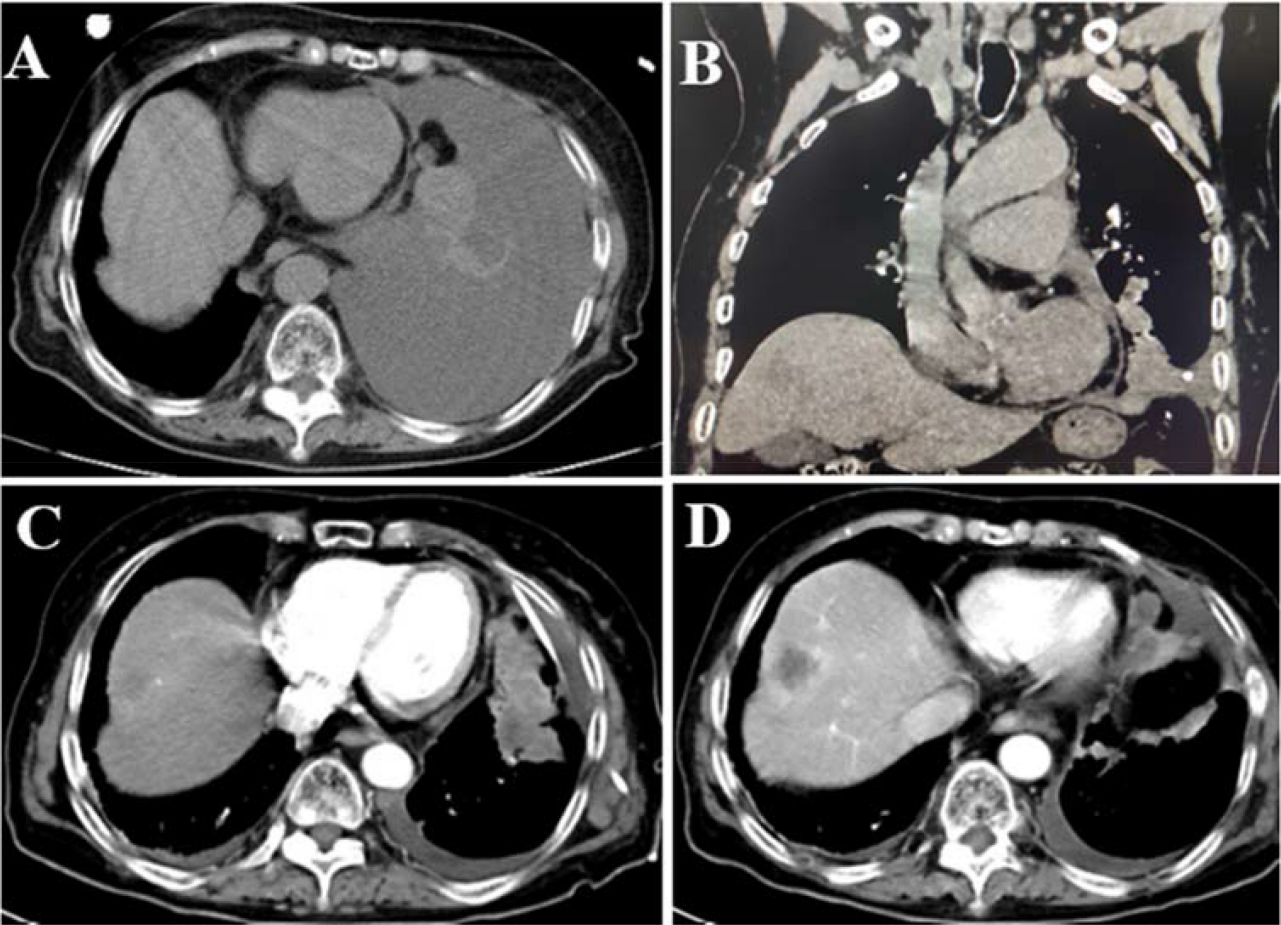
CT images of the reported case. **(A)** Massive left pleural effusion was observed during the initial visit. **(B–D)** Enhanced CT scans of the chest after pleural effusion drainage clearly demonstrated both the lung tumor, pleural metastasis and "bull's-eye" hepatic metastases.

An 81-year-old female with no history of smoking or malignancy presented with a 10-day history of progressive chest tightness and dyspnea, which worsened with physical activity. Additional symptoms included cough and back pain. Chest computed tomography (CT) imaging revealed a large left-sided pleural effusion (**Figure 1A**). To alleviate respiratory distress, thoracentesis was performed, and the drained pleural fluid was sent to the Department of Pathology for further evaluation.

Following thoracic drainage, a subsequent chest CT scan revealed a heterogeneous mass in the left inferior lobe, measuring approximately 6.7 cm × 2.8 cm in its largest cross-section. Contrast-enhanced imaging demonstrated irregular enhancement patterns suggestive of a left lung tumor (**Figures 1B, C**). Pulmonary window analysis identified multiple nodular high-density foci scattered across both lungs, raising suspicion for bilateral pulmonary metastases. Mediastinal window assessment further revealed bilateral mediastinal lymphadenopathy and enlarged lymph nodes at the left pulmonary hilum, consistent with lymph node metastasis. An abdominal CT scan detected multiple low-density hepatic nodules of varying sizes, with the largest lesion (2.9 cm × 2.6 cm) located in the right hepatic lobe. Annular enhancement was noted in the arterial phase, while marked hypodensity was observed in the portal venous phase, demonstrating a “bull’s eye sign” characteristic of multiple hepatic metastases (**Figure 1D**). Additionally, an enhanced brain CT identified an irregular heterogeneously enhancing mass within the right frontal lobe, with a maximum cross-sectional area of 2.1 cm × 1.6 cm, consistent with brain metastasis.

**Figure 2:**
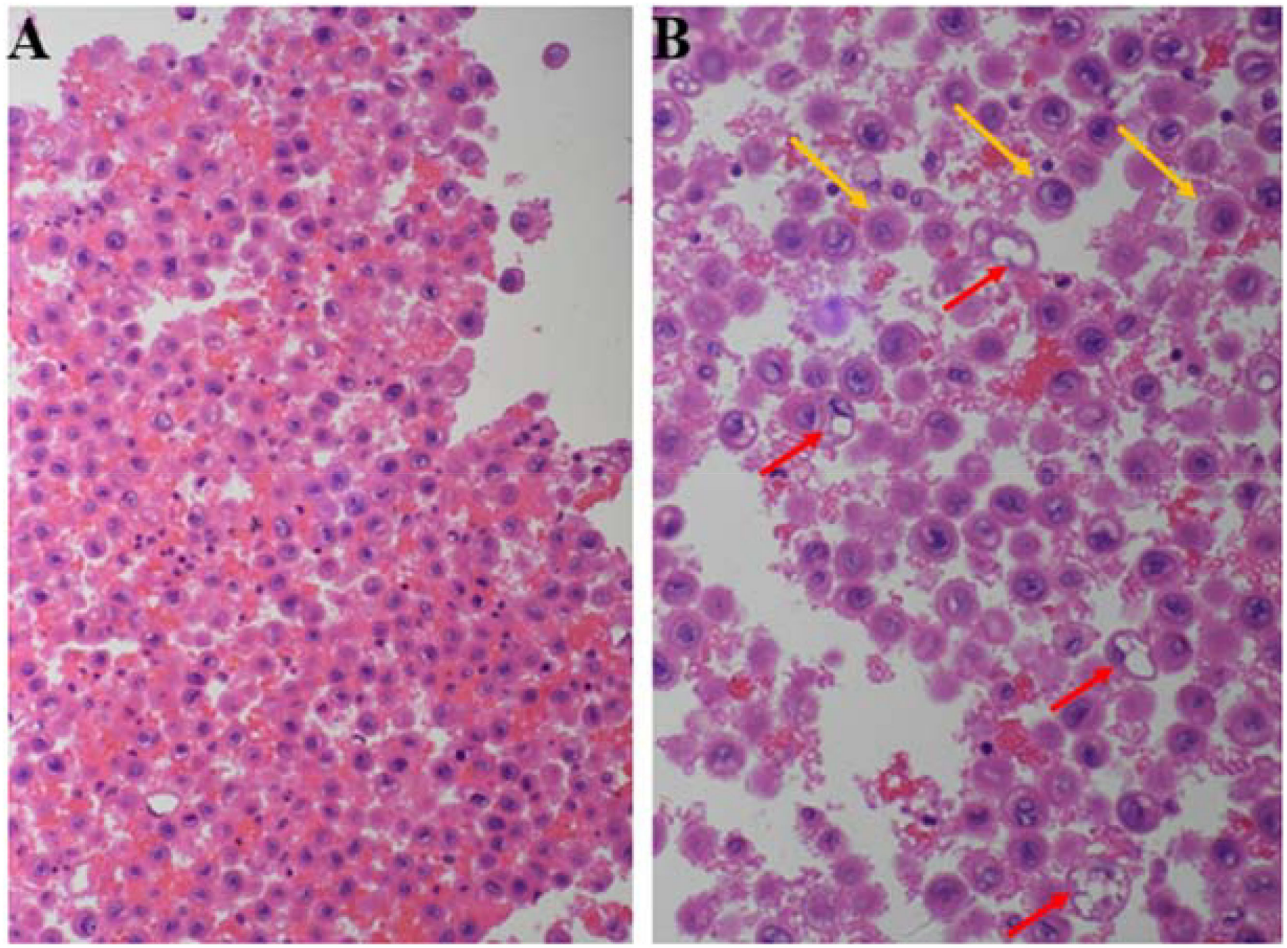
H&E-stained cell block of pleural effusion: round or oval cells with eccentric nuclei and abundant eosinophilic cytoplasm **(A)**. Cells predominantly exhibit rhabdoid morphology (red arrows), with a subset displaying plasmacytoid features (yellow arrows) **(B)**.

Cell block analysis of the pleural effusion demonstrated poorly cohesive round-to-oval cells with a dispersed distribution. These cells exhibited eccentrically located nuclei with dense chromatin, abundant eosinophilic cytoplasm, and nuclear morphology ranging from round to oval or slightly irregular shapes. Mitotic figures were readily identified. The overall cytomorphology revealed a predominantly rhabdomyoid phenotype, characterized by eccentric nuclei and eosinophilic cytoplasm, with focal plasmacytoid features (**Figure 2**).

**Figure 3:**
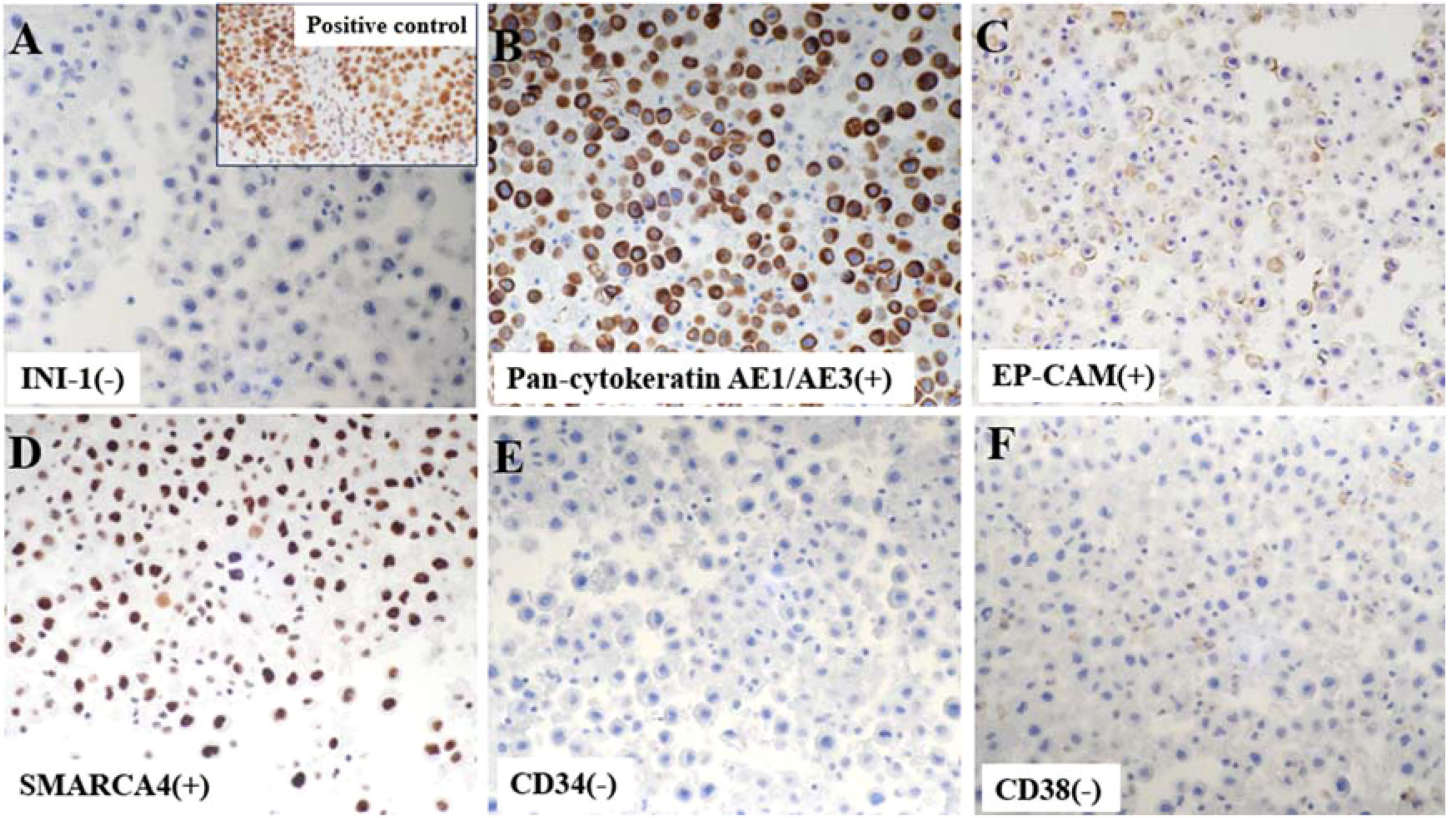
Immunohistochemistry (IHC) demonstrated a complete absence of nuclear SMARCB1 (INI1) expression in tumor cells, with the positive control image from our pathology archive (lung cancer tissue with confirmed INI1 expression) displayed in the upper right corner for reference **(A)**. Tumor cells were positive for cytokeratin AE1/AE3 **(B)** and epithelial marker EP-CAM **(C)** on IHC. Notably, neoplastic cells retained nuclear SMARCA4 expression **(D)**, distinguishing this from SMARCA4-deficient tumors. CD34 **(E)** and plasma cell marker CD38 **(F)** were uniformly negative in tumor cells.

IHC staining revealed strong positivity for epithelial cell adhesion molecule (Ep-CAM), pan-cytokeratin AE1/AE3, and thyroid transcription factor-1 (TTF-1). In contrast, negative staining was observed for calretinin (CR), CD34, CD38, Napsin A, and p40, particularly ruling out malignant mesothelioma, epithelioid sarcoma, plasmacytoma and squamous cell carcinoma. Notably, INI-1 (SMARCB1) expression was completely lost, while SMARCA4 expression remained preserved (**Figure 3**).

Based on the integration of IHC findings and morphological features, a pathologic diagnosis of INI-1-deficient undifferentiated carcinoma was established. Correlating the imaging findings with the pathologic diagnosis, the clinical assessment confirmed metastatic INI-1-deficient undifferentiated carcinoma of the left lung, associated with malignant pleural effusion, bilateral pulmonary metastases, pleural involvement, multiple hepatic metastases, brain metastasis, and extensive mediastinal lymphadenopathy. The disease was classified as clinical stage cT4N3M1c2, corresponding to stage IVB.

NGS-16 panel sequencing identified an EGFR exon 19 canonical deletion (p.E746_A750del, “exon 19 del”), a hallmark activating mutation in NSCLC. No clinically actionable alterations were detected in ALK, MET, BRAF, ERBB2, ROS1, RET, KRAS, NRAS, PIK3CA, NTRK1-3, TP53, MAP2K1, or BIM. PD-L1 IHC (Ventana 22C3 clone) revealed a tumor proportion score (TPS) of <1%.

Follow-up: Given the presence of an EGFR exon 19 canonical deletion (p.E746_A750del, “exon 19 del”)—a sensitizing mutation strongly predictive of response to third-generation EGFR-tyrosine kinase inhibitor (EGFR-TKI) therapy—and concomitant central nervous system metastases, osimertinib was initiated as first-line treatment. Despite commencing oral osimertinib (80mg *QD*) in a community setting, disease progression was observed. The patient, an 81-year-old with a poor performance status and extensive metastatic burden, succumbed to progressive disease one-month post-diagnosis, likely due to the combined effects of frailty and widespread metastasis.

## Discussion

This case represents a rare instance of an INI-1-deficient undifferentiated neoplasm identified in pleural effusion, characterized by monotonous, poorly cohesive cells with eccentric nuclei and rhabdoid/plasmacytoid features—morphologic hallmarks of SWI/SNF complex-deficient malignancies. As highlighted by Reference (12), carcinomas and mesenchymal tumors with SWI/SNF dysfunction frequently exhibit a discohesive epithelioid or rhabdoid phenotype, necessitating a targeted immunohistochemical evaluation of INI-1 (SMARCB1) and BRG1 (SMARCA4) during diagnostic workup. In this case, IHC demonstrated complete loss of nuclear INI-1 (SMARCB1) expression, while BRG1 (SMARCA4) was retained, aligning with the immunohistochemical profile of SMARCB1-deficient tumors. Complete INI-1 (SMARCB1) loss has been implicated in both pediatric and adult mesenchymal tumors, with pediatric malignant rhabdoid tumors (MRTs) and epithelioid sarcomas serving as prototypical examples (19). Additionally, INI-1 mutations have been reported in sinonasal carcinoma, gastrointestinal stromal tumors (GIST), pancreatic cancer, and malignancies of the urologic tract (20–26).

In this case, imaging revealed massive left pleural effusion, and following therapeutic drainage, contrast-enhanced CT demonstrated a left lower lobe mass measuring 6.7 cm × 2.8 cm (maximum cross-sectional diameter) with irregular enhancement. To determine histogenesis and pathologic phenotype, IHC was performed on the cell block using a panel that included Ep-CAM, pan-cytokeratin AE1/AE3, CR, CD34, CD38, TTF-1, Napsin A, and p40. IHC revealed diffuse membranous Ep-CAM positivity and cytoplasmic AE1/AE3 immunoreactivity, coupled with a complete absence of CD34, CD38, and calretinin (CR). These findings effectively excluded key differential diagnoses, including epithelioid sarcoma, plasmacytoma, and malignant mesothelioma. Consequently, the final pathologic diagnosis was established as INI-1-deficient undifferentiated carcinoma. Although molecular studies were not conducted to confirm underlying SMARCB1 alterations, emerging evidence underscores INI-1 (SMARCB1) loss, as detected by IHC, as the most defining and consistently reproducible feature of these neoplasms (1, 27). Upon integrating pathological and imaging data, the lesion was diagnosed as metastatic INI-1-deficient undifferentiated lung cancer. Notably, this case mirrors a report by Kazuki Fujita CT et al. (13), where an INI-1-deficient neoplasm was identified in pleural effusion alongside a pulmonary mass. Importantly, their case involved a patient with plasmacytic myeloma, who also exhibited INI-1 deficiency with SMARCA4 retention. Through comprehensive IHC profiling, recurrence of plasmacytic myeloma was ruled out, ultimately leading to the diagnosis of SMARCB1 (INI-1)-deficient intrathoracic neoplasm of the lung.

Due to the extreme rarity of SMARCB1/INI-1-deficient lung carcinoma, a comprehensive literature review identified eight previously reported cases (12–18) (**Table 1**, **Supplementary Materials**). The first two cases, originally described by Haberecker et al., were part of a cohort of nine intrathoracic neoplasms exhibiting INI-1 (SMARCB1) loss. These cases underwent principal component analysis (PCA) of methylation data, which, along with immunohistochemical coexpression of pan-cytokeratin and claudin-4, provided strong evidence of an epithelial lineage (12). A review of these cases did not establish a definitive correlation with smoking history, as nearly half of the patients were non-smokers. Clinically, seven out of nine (77.8%) patients were aged ≥60 years, and seven (77.8%) had tumors measuring ≥5 cm in diameter at diagnosis. Additionally, five (55.6%) presented with distant metastases, including the present case. Notably, most patients were diagnosed at an advanced disease stage (AJCC stage III-IV in seven out of nine cases, 77.8%), a factor strongly associated with poor prognosis and reduced survival (**Table 1**).

**Table 1:** Demographic and clinical characteristics of the reviewed cohort.

Authors	Ref	Patient	Gender	Age (years)	Smoking history (Y/N)	Primary tumor location	Primary tumor size (cm)	Lymphnode metastasis (Y/N)	Site of distant metastasis	cTNM staging	Treatment	Follow-up/months
Haberecke M,Buhler MM,et al.	(12)	1	M	73	Y	Lung, central	5	Y	Liver, adrenal gland	cT2bN2M1c	Neo-and adjuvant CT	DOD/7
		2	M	72	Y	Lung, centra/hilar	9	N	Lung	cT4N0M1a	Adjuvant CT	DOD/53
Zhou YH,Qin S, et al.	(14)	3	M	74	NA	Lung, RUL	3.1	NA	NA	cT2NxMx	RT	DOD/5+
RickardJA, Burr ML,et al.	(15)	4	M	60	N	Lung, LLL	8.6	Y	Liver	cT4N2M1b	Immunotherapy and CT	AWD
Chen J,WangJ	(16)	5	M	61	N	Lung, LUL	8.8	Y	None	cT4N2M0	Neoadjuvant IT+ CT, RT, IT Maintain	NED/4+
Kazuki Fujita CT ,et al	(13)	6	M	77	NA	Lung, RML	5.1	Y	Lung, adrenal gland, pleural	NA	NA	DOD/0.5
Zagni M, Marando A, et al.	(17)	7	M	38	N	Lung, RML	8.5	Y	None	cT4N2M0	CT	DOD/1
Chen X, Wu J, et al.	(18)	8	M	33	N	Lung, RML	3,4	N	Pleura,right pleural effusion	cT2aN0M1a	Immunotherapy and CT, Tazemetostat	DOD/24+
Present case		9	F	81	N	Lung, LLL	6.7	Y	Left malignant pleural effusion, bilateral lungs, pleural, liver, brain	cT4N3M1c	Osimertinib	DOD/1

RUL right upper lobe, LLL left lower lobe, LUL left upper lobe, RML right middle lobe, NA not available, CT chemotherapy treatment, DOD died of disease, NED no evidence of disease, AWD alive with disease.

SMARCB1-deficient lung carcinomas (INI1 loss) rarely exhibit EGFR mutations. However, according to the 2025 NCCN guidelines, routine EGFR mutation testing is recommended for advanced NSCLC. Following these guidelines, this patient underwent NGS using a 16-gene panel (NGS-16). The results identified a canonical EGFR exon 19 deletion (c.2235_2249del, p.E746_A750del) with a variant allele frequency (VAF) of 57.7%, indicative of a highly clonal mutation. Notably, no EGFR copy number alterations were detected (copy number = 2.0), and the NGS-16 panel ruled out co-occurring oncogenic drivers, including ALK/ROS1 fusions, MET/ERBB2 amplifications, BRAF/KRAS/PIK3CA mutations, NTRK fusions, and TP53/BIM alterations. The coexistence of SMARCB1 loss and a high-frequency EGFR exon 19 deletion (VAF 57.7%) challenges the previous assumption that INI1-deficient tumors are exclusively EGFR wild-type. Although CNV alterations were absent, the presence of a canonical EGFR exon 19 deletion at 57.7% VAF strongly supports the use of EGFR-TKI therapy, as this mutation profile is highly predictive of therapeutic responsiveness. One possible explanation for TKI efficacy in the absence of copy number alterations is that SMARCB1 loss-driven clonal dominance functionally compensates for reduced gene dosage, thereby maintaining TKI sensitivity. This hypothesis proposes a novel mechanism of therapeutic vulnerability in chromatin-remodeling-deficient tumors. In a study, de Miguel, Gentile, et al. demonstrated that the mammalian SWI/SNF chromatin-remodeling complex contributes to osimertinib resistance in EGFR-mutant lung cancer models by modulating chromatin accessibility—a process that enhances the expression of resistance-associated genes and promotes cancer cell proliferation. Importantly, both pharmacological and genetic suppression of SMARCA4/2 successfully reversed osimertinib resistance (9). Based on these findings, this study hypothesizes that INI1 loss, a key subunit of the mSWI/SNF complex, could similarly counteract osimertinib resistance, potentially leading to improved survival outcomes in EGFR-mutant patients. However, further experimental validation is required to substantiate this hypothesis.

As demonstrated in this case, IHC assessment of INI1 expression is crucial for accurately diagnosing INI1-deficient tumors, particularly in tissues with rhabdoid morphology, to prevent missed or incorrect diagnoses. Although INI1-deficient lung cancer is rare, the increasing number of reported cases suggests that it may eventually be recognized as a distinct molecular entity, similar to SMARCA4-deficient thoracic tumors.

While INI1-deficient lung cancer rarely coexists with EGFR, ALK, or other oncogenic driver mutations, comprehensive genetic profiling (CGP) remains essential for guiding treatment decisions, particularly in advanced-stage patients. By providing a detailed molecular characterization, CGP enables a deeper understanding of disease mechanisms, such as osimertinib resistance, and directly supports precision oncology strategies—including the selection of TKIs for EGFR exon 19-mutant tumors. However, the clinical implementation of CGP faces several key challenges, including gaps in evidence-based treatment guidelines, high costs associated with genetic testing, and barriers to interdisciplinary collaboration. Overcoming these obstacles will require coordinated efforts among multiple stakeholders, the establishment of standardized workflows, and the development of policy frameworks to facilitate the translation of genomic insights into clinical practice and maximize patient benefit.

## Data Availability

The datasets presented in this study can be found in online repositories. The names of the repository/repositories and accession number(s) can be found in the article/supplementary material.

## References

[1] Hollmann TJ Hornick JL. INI1-deficient tumors: diagnostic features and molecular genetics. Am J Surg Pathol. (2011) 35:e47–63. doi: 10.1097/PAS.0b013e31822b325b 21934399

[2] Kohashi K Oda Y. Oncogenic roles of SMARCB1/INI1 and its deficient tumors. Cancer Sci. (2017) 108:547–52. doi: 10.1111/cas.13173 PMC5406539 28109176

[3] Clapier CR Cairns BR. The biology of chromatin remodeling complexes. Annu Rev Biochem. (2009) 78:273–304. doi: 10.1146/annurev.biochem.77.062706.153223 19355820

[4] Wilson BG Roberts CW. SWI/SNF nucleosome remodellers and cancer. Nat Rev Cancer. (2011) 11:481–92. doi: 10.1038/nrc3068 21654818

[5] Gonzalez-Perez A Jene-Sanz A Lopez-Bigas N. The mutational landscape of chromatin regulatory factors across 4,623 tumor samples. Genome Biol. (2013) 14:r106. doi: 10.1186/gb-2013-14-9-r106 24063517 PMC4054018

[6] Kadoch C Hargreaves DC Hodges C Elias L Ho L Ranish J. Proteomic and bioinformatic analysis of mammalian SWI/SNF complexes identifies extensive roles in human Malignancy. Nat Genet. (2013) 45:592–601. doi: 10.1038/ng.2628 23644491 PMC3667980

[7] Mittal P Roberts CWM. The SWI/SNF complex in cancer - biology, biomarkers and therapy. Nat Rev Clin Oncol. (2020) 17:435–48. doi: 10.1038/s41571-020-0357-3 PMC8723792 32303701

[8] Centore RC Sandoval GJ Soares LMM Kadoch C Chan HM. Mammalian SWI/SNF chromatin remodeling complexes: emerging mechanisms and therapeutic strategies. Trends Genet. (2020) 36:936–50. doi: 10.1016/j.tig.2020.07.011 32873422

[9] de Miguel FJ Gentile C Feng WW Silva SJ Sankar A Exposito F. Mammalian SWI/SNF chromatin remodeling complexes promote tyrosine kinase inhibitor resistance in EGFR-mutant lung cancer. Cancer Cell. (2023) 41:1516–1534.e9. doi: 10.1016/j.ccell.2023.07.005 37541244 PMC10957226

[10] Nicholson AG Tsao MS Beasley MB Borczuk AC Brambilla E Cooper WA. The 2021 WHO classification of lung tumors: impact of advances since 2015. J Thorac Oncol. (2022) 17:362–87. doi: 10.1016/j.jtho.2021.11.003 34808341

[11] Nambirajan A Singh V Bhardwaj N Mittal S Kumar S Jain D. SMARCA4/BRG1-deficient non-small cell lung carcinomas: A case series and review of the literature. Arch Pathol Lab Med. (2021) 145:90–8. doi: 10.5858/arpa.2019-0633-OA 33367658

[12] Haberecker M Bühler MM Mendieta AP Guggenberger R Arnold F Markert E. Molecular and immunophenotypic characterization of SMARCB1 (INI1) - deficient intrathoracic Neoplasms. Mod Pathol. (2022) 35:1860–9. doi: 10.1038/s41379-022-01133-4 PMC9708576 35864317

[13] Fujita K Mizuguchi K Mori T Shimoda T Sakano K Shimaguchi C. SMARCB1/INI1-deficient intrathoracic neoplasm with rhabdoid/plasmacytoid cytomorphology in a patient with plasma cell myeloma: A case report. Diagn Cytopathol. (2023) 51:E294–300. doi: 10.1002/dc.25197 37475580

[14] Zhou YH Qin S Yan JX Ji J Lan T Liu Y. INI1 (SMARCB1) deletion of lung cancer: report of a case. Zhonghua Bing Li Xue Za Zhi. (2022) 51:902–4. doi: 10.3760/cma.j.cn112151-20220207-00076 36097913

[15] Rickard JA Burr ML Williams B Murugasu A Fellowes A John T. SMARCB1/INI1-deficient primary lung carcinoma with hepatic metastasis. Pathology. (2022) 54:817–20. doi: 10.1016/j.pathol.2021.11.010 35177248

[16] Chen J Wang J. STK11 loss and SMARCB1 deficiency mutation in a dedifferentiated lung cancer patient present response to neo-adjuvant treatment with pembrolizumab and platinum doublet: A case report. Front Oncol. (2023) 13:1088534. doi: 10.3389/fonc.2023.1088534 36776287 PMC9911826

[17] Zagni M Marando A Negrelli M Lauricella C Motta V Paglino G. SMARCB1/INI1-deficient undifferentiated tumour of the thorax: a case report and review of the literature. Pathologica. (2024) 116:163–9. doi: 10.32074/1591-951X-955 PMC11447651 38979590

[18] Chen X Wu J Pang G Wei S Wang P. Integrase interactor 1 (INI1) deficiency in a lung cancer patient presents nonresponse to immunotherapy and tazemetostat: A case report. Cureus. (2023) 15:e42934. doi: 10.7759/cureus.42934 37667707 PMC10475322

[19] Versteege I Sévenet N Lange J Rousseau-Merck MF Ambros P Handgretinger R. Truncating mutations of hSNF5/INI1 in aggressive paediatric cancer. Nature. (1998) 394:203–6. doi: 10.1038/28212 9671307

[20] Agaimy A Hartmann A. SMARCB1(INI1)-defizientes Nierenzellkarzinom: medullär und darüber hinaus: Neue Konzepte [SMARCB1(INI1)-deficient renal cell carcinoma: medullary and beyond: Evolving concepts. Pathologe. (2021) 42:571–7. doi: 10.1007/s00292-021-00985-y 34609565

[21] Agaimy A Hartmann A Antonescu CR Chiosea SI El-Mofty SK Geddert H. SMARCB1 (INI-1)-deficient sinonasal carcinoma: A series of 39 cases expanding the morphologic and clinicopathologic spectrum of a recently described entity. Am J Surg Pathol. (2017) 41:458–71. doi: 10.1097/PAS.0000000000000797 PMC5354087 28291122

[22] Pawel BR. SMARCB1-deficient tumors of childhood: A practical guide. Pediatr Dev Pathol. (2018) 21:6–28. doi: 10.1177/1093526617749671 29280680

[23] Mobley BC McKenney JK Bangs CD Callahan K Yeom KW Schneppenheim R. Loss of SMARCB1/INI1 expression in poorly differentiated chordomas. Acta Neuropathol. (2010) 120:745–53. doi: 10.1007/s00401-010-0767-x 21057957

[24] Agaimy A Rau TT Hartmann A Stoehr R. SMARCB1 (INI1)-negative rhabdoid carcinomas of the gastrointestinal tract: clinicopathologic and molecular study of a highly aggressive variant with literature review. Am J Surg Pathol. (2014) 38:910–20. doi: 10.1097/PAS.0000000000000173 24503755

[25] Agaimy A Haller F Frohnauer J Schaefer IM Ströbel P Hartmann A. Pancreatic undifferentiated rhabdoid carcinoma: KRAS alterations and SMARCB1 expression status define two subtypes. Mod Pathol. (2015) 28:248–60. doi: 10.1038/modpathol.2014.100 25103069

[26] Gupta S Albertson D Gaston D Heilbrun ME Agarwal N Boucher K. Comprehensive genomic sequencing of urothelial tumors identifies rare SMARCB1 (INI-1)-deficient carcinomas of the urinary system. Clin Genitourin Cancer. (2018) 16:e373–82. doi: 10.1016/j.clgc.2017.09.001 28974397

[27] Judkins AR. Immunohistochemistry of INI1 expression: a new tool for old challenges in CNS and soft tissue pathology. Adv Anat Pathol. (2007) 14:335–9. doi: 10.1097/PAP.0b013e3180ca8b08 17717433

